# Supplementary material for: A qualitative analysis of the COVID-19 vaccination rollout in Lagos, Nigeria: Client and provider perspectives on the plan, the process and the progress
Source: PLOS Glob Public Health. 2022 Nov 8;2(11):e0000486. doi: 10.1371/journal.pgph.0000486 (PMC10021997; doi:10.1371/journal.pgph.0000486)
Supplement: Attachment — Submitted filename: Response to reviewers.doc [file pgph.0000486.s001.doc]

**Response to Reviewers**

**Title of manuscript: A qualitative analysis of the COVID-19 vaccination rollout in Lagos, Nigeria: Client and provider perspectives on the plan, the process and the progress**

| Reviewer/section. | Original comments of the reviewer | Reply by the author(s) | Correction made |
| --- | --- | --- | --- |
| **Reviewer #1** | Overall, this paper focuses on the COVID-19 vaccination rollout in Lagos from the perspectives of vaccine recipients, HCWs, and government officers. Overall, the authors did a great job providing and presenting a lot of data on the rollout; I particularly like the inclusion of different perspectives. Some of the language can be cleaned up throughout, but overall, I think it’s a paper the authors should be proud of and is one that will benefit future vaccine rollouts in Nigeria and elsewhere. | The reviewer’s comments are appreciated.  All corrections have been made. | **Corrected,** details below |
| Introduction | Provide a little more detail – perhaps the number of vaccines currently produced (can say “at the time of writing”) to provide context of scale | This has been done. | At the time of writing, the number of doses of the COVID-19 vaccines secured globally was given as 17.5 billion.^[1]^ |
|  | - Lines 80-84: Can you be more specific? Is this clinical data? Consider breaking the sentence into two separate ones for clarity. | The sentence has been fragmented and rephrased for clarity. | The careful process of facilitating the vaccination response against COVID-19 included carefully examining the data available from many parts of the world about the vaccine. This was necessitated by the reports that the use of the AstraZeneca vaccine had been interrupted in many countries, mostly in Europe, as a precautionary measure because some recipients of the vaccine were said to have experienced blood clots as a side effect.^[2]^ |
|  | - Line 84: were the blood clots discredited, or was it reports that it was unsafe? Please clarify. | This has been clarified. | However, these reports about the formation of blood clots in recipients of the vaccine have since been discredited.^[3],^ ^[4]^ |
|  | - Line 85: Is it possible to be more specific than “Africa”? Maybe “Vaccination against COVID-19 has been slower in countries relying on doses from COVAX than in other places” or something like that. | The intention here is to examine the COVID 19 vaccine situation in Africa. | Vaccination against COVID-19 has been slow in Africa, especially for the countries relying on doses from the COVAX  initiative |
|  | - Lines 88-89: This is a very important point, but the sentence is unclear. What proportions are you speaking of? | This sentence refers to the proportions of global vaccines received in Africa. | Africa currently has received only 1%. Africa faces the overwhelming threat of being left behind as the proportions of the global COVID-19 vaccine doses reciveded in Africa seem to be reducing. |
|  | Line 90: An example of an exemplary rollout would be helpful. | This has been corrected |  |
|  | Lines 91-95: This sentence needs some citation(s), as there have been several reports about why the rollouts/administration may have been delayed – including, but not listed here, countries receiving vaccines right before they were to expire. | This has been corrected. | Delays in the complete rollout and administration of vaccines have been attributed to factors such as inadequately trained personnel, a lack of funds to support the vaccination programme, poor planning and vaccine hesitancy based on myths and misinformation, Error: Reference source not found although the apparently slow rollouts may be due to incorrect reporting. |
|  | - Line 96: Which vaccine was provided? | AstraZeneca/Oxford vaccine | In early March 2021, Nigeria received close to 4 million doses of the AstraZeneca/Oxford vaccine, COVID-19 vaccine manufactured by the Serum Institute of India (SII), through the COVAX facility, the vaccine coordinating arm of the Access to COVID-19 Tools (ACT) Accelerator. |
|  | - Line 106: Did Lagos State receive their share immediately? | This has been included. | The Coronavirus vaccination campaign in Lagos State, Nigeria began on the 12th of March 2021 after receiving over 500,000 doses of the AstraZeneca vaccine, ten days after the vaccines provided for use in Nigeria were available for distribution to the States. |
|  | - Line 124: When did the vaccine become available to all adults? | The exact dates have not been identified following my thorough search of the available sources. The search for this date will continue. |  |
|  | - COME BACK TO AIM/LINE 125 | This has been included. |  |
| Methods | Overall, the divisions and sub-divisions are a bit unclear. Consider combining some sections or making note of how sub-sections may fit into broader sections (using numbers and/or letters). | This is noted with thanks. |  |
|  | Line 134: Lagos city or Lagos state? | Lagos State |  |
|  | TOO MUCH INFO? Perhaps link to rollout more and cut the rest… And then connect with study sites | The details are there to give a background of how the health system operates in Lagos State to give a background to the level of care at which vaccination was conducted. |  |
|  | Lines 158-162: What does it mean to be formally approached? How was that different than approaching vaccine recipients? | This has been improved upon. | The interviewees and key informants were formally approached, by prior request via a letter, to be interviewed. |
|  | Data collection tools and techniques: I’m a little confused by “qualitative inquiry.” You mean the qualitative data were collected using a guide, correct? Was this guide structured or semi-structured? Please provide a little more detail on the key topics addressed in the guides and how they differed between IDIs and KIIs. Same can be said for the FGDs – please provide a little information on key topics covered. | “qualitative inquiry” describes the interviews. Yes, the qualitative data were collected using a guide. The guide was unstructured to allow the interviewees provide insights into the vaccination situation that would otherwise be missed. | For the qualitative inquiry, an unstructured moderator’s guide was used to explore the providers’ perspectives on the vaccination roll out plan, and process and progress through in-depth interviews (IDIs) and key informant interviews (KIIs). and The qualitative data about the experiences of vaccine recipients during the vaccination process and their understanding of the vaccine rollout phases were collected through focus group discussions (FGDs) moderated using an FGD guide. |
|  | Where FGDs held the same day as the recruitment of participants? Or did participants return on a separate day? | Yes, the FGDs were held the same day as the recruitment of participants. | Eight (8) focus group discussions, with 8 vaccine recipients in each group, were carried out in each of the selected PHCs. The discussions were held soon after the participants received the vaccine, on the same day of vaccination, in adequately spaced rooms or open spaces that provide for privacy, |
|  | Line 180: The number of IDIs, etc. carried out should be included in the results section. Also, if there were 8 sites and 4 IDIs were carried out at each site, why were 24 conducted (rather than 32)? 24 is still a lot of IDIs, but please comment if there were decliners or a reason why the numbers do not match. | The number 4 was written in error. This has been corrected. | Three (3) in-depth interviews were conducted in each of the selected Local Government Areas. Among the 3 front-line health workers ((a) the Local Government immunization officers, (b) the disease surveillance and notification officers and (c) the medical officers of health). In all, 24 IDIs were conducted. |
|  | Lines 183-186: Is there a way to not include their titles? I imagine this makes the KII participants easy to identify… Please be more general in how they are referred to. | Thank you for this observation. The authors agree with using identifiers. | Three (3) in-depth interviews were conducted in each of the selected Local Government Areas among the 3 main front-line health workers in PHC. In all, 24 IDIs were conducted.  Three (3) key informant interviews were conducted among the 3 key officers in the Lagos State Ministry of Health. |
|  | Lines 191-192: Please clarify. “Transcribed verbatim” means word-for-word, so by the rest of the sentence, do you mean that the other notes (e.g. non-verbal cues, etc.) were included into the transcripts as well? | Yes, it means word-for-word transcription, bit probably because of the topic of discussion participants. Non-verbal cues were mostly insignificant. However, some mannerisms that come with the mode of expression of people in southern Nigeria were recorded such as the occasional ‘’ah!” or “oh”, especially where it bore an important meaning like displeasure or affirmation. |  |
|  | Line 196: “manuscripts” should be changed to “transcripts” | Done | The authors read the transcripts several times. |
|  | Lines 199-207: There is a lot of detail here that I believe can be written/edited in a way that is more succinct and clearer to the reader. | This has been edited. | First, the verbatim transcripts were read and reread for overall understanding, and so that the authors familiarized themselves with the details of the transcripts. The transcripts were, then examined and coded to search for patterns (content analysis) based on a theoretical framework, identifying relationships and similar phrases. |
|  | If no coding software was used, did the authors use Word or Excel | The authors prepared the transcript in Word. |  |
|  | The number of KIIs, IDIs, FGDs should be included at the beginning of the results section. Here you can also include some summarized information on the participants (gender, age, etc.) | These have been included. | In this study 8 FGDs, 24 KIIs and 3 IDIs were conducted. The FGD participants were all adults, mostly female (75.0%), male-female ratio 1:3. |
|  | -Were men and women included in the same FGDs?  - What is the gender breakdown of the IDIs and KIIs?  - I’m still worried that by providing their titles, the identity of the KIIs is easy to figure out. | Yes  They were not selected based on their person but on their official roles as frontline workers.  The titles have been removed to mask their identities. | Eight (8) focus group discussions, with 8 vaccine recipients in each group, were carried out, one in each of the selected PHCs. Men and women included in each FGD. |
|  | - Lines 252-254: did the participants themselves have concerns about the government’s motives or the uncertainty of the effect, or was it what they had heard from others? If they had these concerns, what led them to still get vaccinated? | The participants were expressing their opinions of others. Those who came forward to receive the vaccines did not have these concerns |  |
|  | - In this section (myths/misconceptions), it would be helpful to have a specific example or two. Participants seem to have heard of the myths (did they mention any specific myths?) but still got vaccinated. Did anyone say why that was? | The clients did not give examples but were referring to the misinformation that was being propagated. |  |
|  | OK, this is mentioned in the next section. Was it the same respondents? | Yes |  |
|  | - Lines 303-305: Was this hesitancy the HCWs had seen while working in the clinic – was it directly observed by them? Or was this more of an explanation for wider vaccine hesitancy? | They were referring to the situation in and outside the clinic regarding vaccine hesitancy. The health workers worked in the clinic and in the community. Part of their work was to enlighten the people in the community during outreaches. |  |
|  | Lines 308-311: Were participants asked to score the programme during the IDIs? Was this a formal assessment, or was it part of the interview questions? If it was formal, please say more on that and include it in the methods section. | The participants were asked to score the process on a scale of 1 to 5 as part of the interview questions. It was not a formal scoring. |  |
|  | Line 323: should there be “not” in that sentence? | In normal parlance it should read *“not supplied enough of these things”,* but considering his use of local speech mannerisms the participant meant that they were supplied but without consideration for how much of them would be needed. | *“I think we didn't pay enough attention to the logistics that were going to be involved, when I say the logistics am talking about down from the issue of the cotton wool that will be used, the gloves that will be used because we were supplied these things* {*not supplied enough of these things}.* |
|  | Lines 339-351: Since the steps are already mentioned in the intro, I think this section can be cut. Or, the focus should not be on repeating the steps, but on the KII participants’ views on how this went, barriers/successes, etc. | This comment is much appreciated. | This aspect adds in a little more detail as would be expected form the executors of the vaccine rollout plan. Omitting this section might appear that the key informants did not know the plan. By this the affirm that they followed the plan. |
|  | Section from KIIs in general, lines 331-461: I have a few concerns about this section.  o First, there is a lot of space given to what was only 3 KIIs in comparison to the IDIs and FGDs. Much of the first part is just a summary of the process that occurred, much of which is included in the intro. I think what is most relevant for the study can stay in the intro and then the rest can be removed and instead placed in a figure or text box, possibly to be included in the annex or as supplementary information. | The Lagos state COVID-19 roll out plan was executed under the direct purvue of the 3 key officers interviewed in the KIIs. Thus there could only be 3 KIIs.  The suggestion of s text box is excellent and very well received. |  |
|  | The parts that bring up the KII participants’ opinions on what went well and what can be improved upon are good and could be elaborated on – especially as their comments relate and compare to what the IDI participants said. | This is well noted. |  |
|  | Lines 423-425: is this the opinion of the participants? As it reads, it sounds almost like they are summarizing a study they did. | This has been edited for clarity. | Vaccine uptake was reported as extremely low in some centres. The poor turnout for vaccination was perceived by key informants to be linked to poor awareness. They noted that the widespread vaccine hesitancy |
|  | I’m not sure what is different between “barriers” and “challenges.” | Barriers here referred to those factors that crippled the rollout plan especially fron the perspective of the provider, while challenges refer to factors that made the process less seamless especially from the perspective of current and prospective clients. |  |
|  | - In general, the sub-sections of the Results all follow the same order of plan, process, and progress for each group of participants separately. Something to consider would be blending the results from the different participants so they can be compared and contrasted as it relates to this overall main outline (plan, process, progress). This would keep make the results more succinct, allow for comparison between different groups of respondents, and make the sections and sub-sections easier to follow. | This is noted and very much appreciated. However the intention of the authors is to highlight the perspectives at different levels so as to inform future interventions. It is hoped that presenting the results in this way would allow measures to be designed and applied specific to the clients and/or providers. |  |
|  |  |  |  |
|  | - Lines 480-481: Sentence needs to be written more clearly. | This has been adjusted for clarity. | Some studies have suggested that vaccine hesitancy may be particularly prevalent in low-income countries like Nigeria, where our study was conducted, precipitated by factors such as distrust in vaccines. |
|  | Lines 502-507: Are there other reasons why certain sites had better turnout than others? Was it only do to misinformation, or are there structural factors that may play a role? | The respondents did not provide any other explanations to the poor turnouts. |  |
|  | Lines 507-508: Citation? |  | Pugliese-Garcia M, Heyerdahl LW, Mwamba C, et al. Factors influencing vaccine acceptance and hesitancy in three informal settlements in Lusaka, Zambia. Vaccine. 2018;36(37):5617-5624. doi:10.1016/j.vaccine.2018.07.042 |
|  | Lines 513-516: Can you be more specific? Perhaps include more of the information participants gave regarding how or what they want included in vaccine messages and how the rollout may address those issues in the future. | The participants gave a few suggestions already stated in the manuscript. | …such as in their local language or using tailored language that they can easily understand |
|  | Lines 517-520: I’m not sure I understand this sentence… How does high morbidity/mortality lead to a reduction in vaccine hesitancy? Also, this is a rather old source – is there anything newer that addresses this issue, particularly as it may relate to COVID? | Because the disease has a high morbidity and mortality more people accept to receive the vaccine to save themselves fron ill health and death. | Other studies investigating predictors of vaccination uptake, and factors associated with poor vaccination coverage found that the perceived severity in terms of the high morbidity and mortality of a disease such as COVID-19 may reduce vaccine hesitancy,^38^ demonstrating that vaccine uptake is highest before and during a pandemic, and immediately after a new vaccine is available to control the pandemic. ^39^ |
|  | Lines 526-531: Is it the AEFIs themselves, or people’s perceptions of the AEFIs that is most important for vaccine uptake? | The participants identified AEFIs as an enabler of vaccine hesitancy. |  |
|  | Lines 536-541: I find that participants wanted a more bottom-up approach interesting. Was this something that the KII participants discussed too? Is there a way to compare/contrast this desire with the plans of the rollout? | The KII participants did not proffer this as a solution. The problems of the rollout plan brought about the recommendation of a more bottom up approach. |  |
|  | Lines 542-548: Have there been any studies about incorporating other vaccines into routine immunization? | As much as the authors know, no study has been done |  |
|  | - Lines 552-556: Can you provide an example of the use of these types of partnerships? How would COVID vaccine rollout be similar or different from these examples? Are there importance lessons that can be applied? | This is noted. Additions made. | This opinion may have been influenced by the numerous examples of the successful interface of the government and private organizations in healthcare provision in many settings in Nigeria^[5],[6]^ and globally. ^[7],[8]^ COVID vaccine rollout would be similar to these examples in the sense that the limitations of poor funding would be eliminated. |
|  | - Overall, how can other countries learn from Nigeria? This study gives details on many challenges and lessons learnt – how can other countries benefit from this information? How can Nigeria benefit from this information in the future? |  |  |
| **Reviewer #2** | This paper reports on a qualitative study that assessed the plan, process, and progress of the COVID-19 vaccination program in Lagos State Nigeria. Using in-depth interviews and focus group discussions, the authors highlight, from the perspectives of vaccine recipients and healthcare workers, the challenges to the COVID-19 vaccine implementation program in Lagos State including issues around vaccine hesitancy and logistics. Factors that contributed to the success of the program are also presented. The study is novel and has interesting and important findings that could be used to inform future vaccine programs in Lagos and Nigeria generally | The comment is appreciated |  |
|  | The presentation of the paper however needs to be improved in terms of structure, additional information in the Method’s section, and support (quotes from IDIs and FGDs) for some of the key results. | This is noted. |  |
|  | Going through the results and certain sections of the abstract, it became less and less clear if the manuscript is reporting on the outcome of research or a monitoring and evaluation exercise. Perhaps this is because of the way the results are presented (especially the choice of words). For example, participants agreed, commending the government of Nigeria, and detailed descriptions of the actual roll out program instead of presenting the perspectives of the different stakeholder groups without the authors qualifying them as: “genuine” “successful, “unfounded claims” etc. Also, where the authors present the results of IDIs with healthcare workers and key informants, there is an inclination to provide a defence to most of the challenges mentioned by the interviewees. That may be okay, if those reasons are provided in the discussion section for example… although in the interviews, participants mentioned that XXX was a major logistic-related challenge, from our knowledge and experience of the roll out program, this challenge may have been due to XX and was resolved by XX. | This is noted. The presented opinions are those of the participants. The language has been adjusted throughout the paper for clarity. |  |
|  | Following up from the point above, it is important for the authors to describe their positionality in the context of this research. For example, were some of them involved in the vaccine roll out program and if so in what capacity and how this may have influenced the results or discussions? This will give researchers some perspectives on the overall narrative and arguments advanced in the paper. | The authors all work in the health sector in Lagos State and hence are all involved in the COVID-19 response in various capacities. However none work in the excact capacity as those interviewed. The results were presented without any influence of who the authors are. |  |
|  | The methods section needs more information: | This is noted with thanks. |  |
|  | Line 159: vaccine rollout process or vaccine roll out program? | Either word is suitable | The study participants were purposively selected based on their involvement in the vaccine rollout programme |
|  | • Line 160: It seems to me like two sampling strategies were used. Purposive (frontline workers) and convenience (clients/vaccine recipients). | At the vaccination sites other clients who may have been yet to to receive the vaccine of who were there for other reasons were present, but only thode who had just received their vaccine were purposively recruited for the study. |  |
|  | Line 161: Participants were formally approached- What does this mean? -A description of the process and why that was necessary will provide more clarity | ‘formally approached’ here means that they were written formal letters requesting their availability to be interviewed for the study. | The interviewees and key informants were formally approached, by prior request via a letter, to be interviewed. |
|  | Line 164: Delete “For the qualitative inquiry”. The title already states that it is a qualitative study. Also, only qualitative methods were used | This is noted with thanks. |  |
|  | Line 164: Moderator’s guide: What was the nature of this guide? Semi structured, open ended or close ended? What type of questions were included and how was it developed? |  | The moderator’s guide was produced from a knowledge of already existing problems related to the COVID -19 vaccine roll-out and from previous studies.^[9],[10]^ |
|  | Line 165: State the number of IDIs and KII. What was the difference between the two? was it in the method or in the stakeholder group interviewed? If in methods, then I suggest that should be described. If it is in the stakeholder group, then I suggest you use IDIs across the paper. | The numbers were stated. The difference is in the person interviewed. Key informants are different from the other respondents because of the role they play in the situation under consideration. |  |
|  | The methods section should have a justification of why different methods were used for the different stakeholder groups. For example, why FGDs with vaccine recipients and not IDIs. Similarly, why IDIs with frontline workers and not FGDs. Also, it needs to be stated clearly at the introduction that FGD were conducted with vaccine recipients only and IDIs with healthcare workers and key informants (Administrators?) only. Otherwise, it becomes confusing who was involved in what, especially in the results section. |  | An FGD was preferred over individual interviews for the vaccine recipients so as to gather information beyond participants’ knowledge, attitudes and practice related to the COVID-19 vaccine and also explore their shared attributes and differences, if any, which otherwise would be impossible to examine.^[11]^ The specific frontline workers required for our study in each PHC do not number enough to make up an FGD. |
|  | How long were the IDIs and KIIs? This information was provided for FGDs but not the other methods. | These have been included. |  |
|  | How did the authors arrive at the final sample size for each stakeholder group and in the case of FGDs, why 8 participants per FGDs? | We were guided by the number if providers in each facility  FGDs typically constitute 6-10 people. We chose to work with 8 people since we had to obseve COVID 19 protocols, we considered that participants would be very far apart. |  |
|  | • What was the overall study period? When were the FGDs conducted, was it immediately after vaccination? If yes, how were participants selected from the overall pool of persons who presented at the vaccine site on that specific day? |  | This study was conducted from May to July 2021.  The vaccine recipients were recruited at the vaccination centres right after they had received either the first or second dose of the vaccine, after the study was explained to then and they gave consent. The interviewees and key informants were formally approached, by prior request via a letter, to be interviewed. |
|  | What was the criteria for selecting the key officers? That is how did the team decide who was a “key informant”? for example why the state epidemiologist and not the Commissioner of Health or the Head of the Nigeria CDC? | The selected Key officers where those who were directly in charge of the vaccines and the COVID 19 Response. The Comissioner oversees these officers. Head of the Nigeria CDC oversees the country and not just Lagos state. This study sought to examine the Lagos state situation at its very rudimentary levels. |  |
|  | Line 188: Were all the Investigator part of the training or only those responsible for the conducting the FGDs, KIIs etc If not all, consider indicating the initials of those who were trained for this purpose. |  | The investigators ( KOJ and OY) and research assistants underwent training on conducting FGDs, KIIs and IDIs |
|  | Line 195-196: What does transferability for cultural reasons mean and what impact did this have on the thematic coding and data analysis. | The themes presented were considered and presented based on the interpretation of the responses of the participants in the light of the cultural backround of their responses. For example: (An Exerpt from the transcript, P=participant, I=interviewer)  P: Going forward  I: Okay. Thank you ma. Do you think the covid 19 vaccination would progress to reach every citizen as planned?  P: That is like asking me to chose between the devil and the deep blue sea, I believe in Lagos!  “I believe in Lagos” is the local way of expressing hope in the vaccination process. It is based on the attitude of the current Governor of Lagos and the current government that promotes and encourages progress and success and have maintained a track record for doing great things. This is a known way to communicate among the locals in Lagos and Nigeria. Transferability means that the local parlance is reported in a way that people from any part of the world can relate to it. |  |
|  | • Line 196: Read the manuscript (transcript?) | This has been corrected. | The authors read the transcripts several times |
|  | Line 199: A description of the theoretical framework is important to give readers a perspective of the analysis. Is it an existing framework that has been published or used in similar studies or was it developed during the coding process by those involved in the analysis? | It is an existing framework that has been published or used in similar studies. The reference was provided. |  |
|  | Line 200: The sentence, “Next, by the process of indexing. This process of indexing needs to be described or referenced for the benefit of your readers. | Reference included. | Bloor, M. & Wood, F. (2006). Indexing. In Keywords in qualitative methods (pp. 102-104). SAGE Publications Ltd, https://dx.doi.org/10.4135/9781849209403 |
|  | • Line 203: What is understandable data? Either provide a description or provide a reference of what it means in the context of qualitative research. | This has been rephrased. | This was followed by a summerisation of the findings into simpler, easy to understand sentences |
|  | Line 205: Were the several meetings with the four team members involved in the coding or all the authors. |  | The team was decribed in an earlier sentence that reads, “The transcripts were reviewed by a team of four of the authors (OJK, YO, HA, and AO)”. This was the team that had several meetings. |
|  | • Line 213: Purely voluntary- I suggest purely be deleted except it goes beyond voluntary. | Done |  |
|  | Table 1: Headings in Column 1 are not consistent- In one instance the method is used, in another the name of the stakeholder group. I suggest the authors use 1 across all groups, preferably the stakeholder group (Vaccine Recipients; Front line health care workers etc) | This has been modified |  |
|  | Overall, the presentation of the results is confusing. It is presented according to the Methods. I suggest the headings should rather be defined by the different Stakeholder Groups. For example, “Perspectives of vaccine recipients on the COVID-19 vaccine rollout Program in Lagos”; “Perspectives of frontline healthcare workers on the COVID-19 vaccine rollout Program in Lagos” etc. | This has been modified |  |
|  | Line 234: Introduce a quote from the FGDs to support the statement. |  |  |
|  | Line 245: The theme needs to be rephrased to capture the goal of the project (COVID-19 vaccine roll in Lagos). For example, “Myths or misconceptions about COVID_19 Vaccine and the impact on the vaccine-roll out program in Lagos State”. The paper is on the vaccine roll out process and not COVId-19 vaccine hesitancy. How it leads to hesitancy can then be captured in the narrative rather than the sub-heading (a key theme) | This has been corrected. |  |
|  | • Line 249: A supporting quote should be inserted at the end of the sentence “…… conspiracy theories about theCOVID-19 vaccine” to support the analysis/narrative |  |  |
|  | Line 249: Sentence starting with 'Unfounded claims'…. I suggest that as much as possible, the authors should remain value neutral in presenting the results and avoid the use of words like “unfounded” except that was used by the participants and there is a supporting quote for that. That phrasing, if necessary, can be used in the discussion section with a supporting argument on why they are considered “unfounded”. I think here is an opportunity by the authors to educate, rather than then dismiss a claim as unfounded” because such dismissals could entrench covid-19 vaccine hesitancy or resistance. | The language has been corrected. | According to the clients, the unfounded claims propagated through social media about the harmful nature of the vaccine were the major reason for vaccine hesitancy. |
|  | Line 250: Participants mentioned that..….. Was this all or some, or most of the participants? This applies across the manuscript. | Noted with thanks, Corrections made. |  |
|  | • Line 267. Sentence ending with “achieved faster” needs a supporting quote. Throughout the results, please provide supporting quotes for each result. | Noted with thanks, quotes added. |  |
|  | • Line The statement “It was also opined that vaccination would help improves the economy…” also requires a supporting quote | Noted with thanks, quotes added. |  |
|  | Why are supporting quotes from the IDIs not reported in the same way as the FGDs?  The FGDs for example had the gender and age of the participants in addition to the unique participant number, while the IDIs only have the unique IDs. This needs to be consistent across the paper. Analysis in terms of age and gender are also not presented, so it is hard to tell why the authors reported the FGDs using gender and age. | This was aimed at reducing the length of the paper.  The FGD participants represent the population of Nigeria receiving the vaccine and their demographics in terms of age and gendre were considered important. The IDI and KII participants, male or female, young or old, were selected purely based on their professional roles. |  |
|  | Lines 314-318: Up to three quotes are provided to support the results that HCWs commended the government, and all quotes are very similar. I suggest one quote is used for this purpose especially as in most cases, just one quote is provided for a key result- and in other cases no quotes. | This is noted with thanks. |  |
|  | Lines 320- Kindly provide description on the specific aspect(s) of planning that came up from the interviews before providing a quote. One quote should be okay. | The participants being healthworkers trained and prepared for the rollout knew all about the plan. All aspects of the plan were to be discussed. They expressed the aspect of the plan they wished to highlight. These quotes are provided to provide readers the context of their responses. |  |
|  | Line 332: Considering the length of this manuscript and readers may have forgotten which stakeholder groups were described as “Providers”, It is important, for the purpose of clarity, to repeat that information in this sentence - May be important to repeat it here for clarity. | This has been included. |  |
|  | • Line 339-351: I suggest the paragraph be deleted as it doesn’t form part of the key results nor adds value to the results. Same for like 357-359. | These are included because they are necessary to provide context regarding the fine details of the administrative background of the rollout plan. |  |
|  | Line 367: Please provide a supporting quote. | Noted with thanks, quotes added. |  |
|  | Line 370-71: Why was there a need to review the process? I think that is the key information as the article reports on vaccine roll out process. |  | However, the constituents of a team was reviewed to include two recorders and no town announcers, given that the role of an announcer was no longer required after a few weeks, |
|  | Line 386: Issues around logistics has already been reported. Also, Lines 397-407 seems to be an evaluation of the logistics rather than the perspectives of the key informants. I therefore suggest that the paragraph be deleted. | The writing style has been corrected to reflect that the details are the participants perspectives. |  |
|  | Lines 442: Please provide supporting quote | Noted with thanks, quotes added. |  |
|  | Lines 443: For the entire section, please provide supporting quotes for each of the key results. | Noted with thanks, quotes added. |  |
|  | Lines 450-453: It is not clear how failure to observe COVID-19 protocols by the already vaccinated, was a challenge to the vaccine roll out program in Lagos. That will need further explanation |  | A patient-related challenge to the entire COVID-19 response highlighted by study participants was that despite contrary information, people who were vaccinated let their guards down in terms of observing COVID-19 protocols such as hand washing, social distancing, wearing of face masks |
|  | Lines 469: The word client has only been introduced in the discussion. My guess is that this refers to vaccine recipients. However, I think, the authors should opt for one description for that stakeholder group and use it throughout the manuscript. | This is noted with thanks. Corrections made. |  |
|  | Overall, the use of “participants” across the results section is confusing, I suggest it should be replaced with the title of each stakeholder group that is being referred to. | This is noted with thanks. Corrections made. |  |
|  | Generally, it is not clear why the results were analysed and presented separately for each stakeholder group? Why was that important and what extra value does it bring to the narrative on COVID-19 vaccine roll out in Lagos. This needs to come out clearly in the introduction and discussion especially as some on the themes are similar across stakeholder groups. If there are no specific reasons, the authors should consider reporting the results by thematic area only and supporting quotes should indicate if it was a HCW, key informant or vaccine recipient. | It was important to keep the perspectives separate so as to enable the lessons learnt be exclusive to each level. |  |
| Reviewer #3 | This is a very interesting and important article, outlining in great detail the complexities and successes of delivering COVID-19 vaccination in Lagos. I think the article could state its contribution even more strongly, by noting its originality in bringing together perspectives of vaccine recipients and those involved at different levels of the vaccine delivery system. Emphasising the contribution will also require the authors engaging a bit more thoroughly with the social science literature on vaccine confidence and delivery - the Vaccine Confidence Project (<https://www.vaccineconfidence.org/>) can be a good start, and literature by Heidi Larson and colleagues includes some good overviews of the literature. | This is noted with thanks. |  |
|  | The area where I think some major revisions are required is in the interpretation of the data. This will not require a great deal of re-analysis, but rather really considering what the data is saying and what it is not. It seemed to me that having interviewed vaccine recipients and providers, the authors cannot in fact suggest to have insight into drivers of vaccine hesitancy. Discussions of conspiracies and mistrust are *reported* by respondents but not in fact stated as reasons for *their* refusal. They are in other words speculations about low uptake. For example on page 20 you state “the poor turnout for vaccination was because awareness was low”— but you haven’t actually explored reasons for refusal/hesitancy with those who refused, so you can only really say that poor turnout was perceived by key informants to be linked to low awareness. | This is noted with thanks and has been corrected. | Vaccine uptake was reported as extremely low in some centres. The poor turnout for vaccination was perceived by key informants to be linked to poor awareness. They noted that the widespread vaccine hesitancy |
|  | Research has shown that healthcare providers might overstate low awareness when reasons for hesitancy are more complex (see for example Enria et al "Bringing the social into vaccination research" in PLOS One). It does not mean that the fact that providers and vaccine recipients deem this to be significant is not worth stating, but it needs to be interpreted as their speculation rather than as a finding that this is what you take to be the most important issue affecting vaccination uptake. The respondents' description of their encounters of misconceptions in practice are valuable, but need to be interpreted as such, given that they did not in fact drive their own vaccination behaviour (everyone you interviewed is pro vaccination). Is there any data available on uptake in the facilities you studied and the relative significance of supply and demand issues? On page 27 you note “poor uptake”—can this be quantified? Were any access issues reported in your findings? Is there any research you could cite that offers more concrete evidence on the hesitancy problem, if this is indeed a large problem in practice? I thought that it was extremely interesting that your respondents worried there might not be enough vaccines for everyone—how does this relate to concerns about mistrust? | Comments are noted with thanks. These important insights have been brought to bear throughout the paper. |  |
|  | Related to the question of potential hesitancy, I would suggest that you consider a more nuanced interpretation of mistrust in vaccination. There may be more complex reasons why people have low trust in vaccination, and indeed there is very good literature on this from Nigeria as well, which situates for example the polio controversy (see Obadare E. A crisis of trust: history, politics, religion and the polio controversy in Northern Nigeria. Patterns Prejudice. 2005;39(3):265–84.) or broader literature that contestes the “knowledge deficit” model (see Goldenberg M. Public Misunderstanding of Science?: Reframing the Problem of Vaccine Hesitancy. Perspect Sci. 2016;24(5):552–81.). You can consider this in the discussion, but as noted above, I would also treat the issue of mistrust much more carefully, with a more nuanced interpretation and perhaps not make it your central finding as I am not sure that is what your data suggests. | Comments are noted with thanks. These important insights have been brought to bear throughout the paper. |  |
|  | Because you are reporting data from very different kinds of respondents, I would make sure that throughout you make clear which you are speaking of—so for example on page 20 instead of saying “most of the respondents”, clarify that this is among key informants | This is noted with thanks. Corrections made. |  |
|  | P.5 why is this study framed as a “risk assessment”? | In the introduction the information provided is to provide a background about the study. |  |
|  | How were the 8 sites selected? | This has been written better. | This was conducted by multistage sampling from the list of 88 COVID-19 vaccination sites in Lagos.^[12]^ The LGAs where the COVID-19 vaccine was available were first stratified into urban and rural LGAs. Next, 7 LGAs from the urban LGAs and 1 LGA from the rural LGAs were selected by ballot, a total of 8 LGAs. Also by ballot one vaccination site from each of the 8 selected LGAs was included in the study. Eight (8) sites in all were selected for the study. |
|  | Clarify the categories (i.e. HCWs were also “vaccine recipients” but I assume you are differentiating?  Are there any more demographic characteristics on vaccine recipients?  How were they selected?) | Corrected  Gendre and age only | The study was conducted among clients (vaccine recipients) at the designated COVID-19 vaccination centres  The clients were recruited at the vaccination centres right after they had received either the first or second dose of the vaccine |
|  | You mention using a theoretical framework—what was the framework used? | A theoretical framework used for our research refers to a short summary of the key concepts and theories surrounding vaccines, the rollout plan, vaccine uptake and the factors mitigating against the success of the exercise. It describes any possible relationships between these concepts. The framework is based on a literature review. A strong theoretical framework gives research direction, showing that it is grounded in established ideas, allowing the researcher to convincingly interpret, explain and generalize from their findings. | The theoretical framework summarized the key concepts and theories surrounding vaccines, the rollout process, vaccine uptake and the factors mitigating against the success of the exercise describing any possible relationships between these concepts. The framework is based on a literature review. |
|  | - I would make clear from the beginning that vaccine recipients were interviewed through FGDs, HCWs and Key informants through interviews.  Maybe you could move Table 1 to the methodology so it’s clearer from the start. | This is noted with thanks and has been corrected.  This is noted with thanks, will be ensured with the final proof document. | Data collection was entirely a qualitative inquiry, from the clients through focus group discussions (FGDs), and from the frontline workers and key informants through in-depth interviews (IDIs) and key informant interviews (KIIs) respectively. Data about the experiences of vaccine recipients during the vaccination process and their understanding of the vaccine rollout phases collected through FGDs were moderated using an open-ended, easy to understand, non-judgemental, justifiable, agreeable FGD guide. An unstructured moderator’s guide was used to explore providers’ perspectives on the vaccine rollout plan, process and progress through the IDIs and KIIs. |
|  |  |  |  |
|  |  |  |  |
|  |  |  |  |
|  |  |  |  |
|  |  |  |  |
|  |  |  |  |
|  |  |  |  |
|  |  |  |  |
|  |  |  |  |
|  |  |  |  |

1. Unicef. COVID-19 Vaccine Market Dashboard. Available at: <https://www.unicef.org/supply/covid-19-vaccine-market-dashboard>. (Last accessed: 14 March 2022)
2. World Health Organisation. WHO statement on AstraZeneca COVID-19 vaccine safety signals. Available at <https://www.who.int/news/item/17-03-2021-who-statement-on-astrazeneca-covid-19-vaccine-safety-signals> (Last accessed 9 September 2021)
3. Statement of the WHO Global Advisory Committee on Vaccine Safety (GACVS) COVID-19 subcommittee on safety signals related to the AstraZeneca COVID-19 vaccine [Internet]. [cited 2021 Apr 7]. Available from: https://www.who.int/news/item/19-03-2021-statement-of-the-who-global-advisory-committee-on-vaccine-safety-(gacvs)-COVID-19-subcommittee-on-safety-signals-related-to-the-astrazeneca-COVID-19-vaccine
4. WHO statement on AstraZeneca COVID-19 vaccine safety signals [Internet]. [cited 2021 Apr 7]. Available from: https://www.who.int/news/item/17-03-2021-who-statement-on-astrazeneca-COVID-19-vaccine-safety-signals
5. Jinadu FO, Agunloye AM, Adeyomoye AA, Adekoya AO, Obajimi GO. Public–private partnerships in Nigerian teaching hospitals: Potential and challenges. West Afr J Radiol 2020;27:143-9
6. Okafor C. Improving Outcomes in the Nigeria Healthcare Sector through Public- Private Partnership. African Research Review. 2016; 10(4):1 DOI: 10.4314/afrrev.v10i4.1
7. Joudyian, N., Doshmangir, L., Mahdavi, M. et al. Public-private partnerships in primary health care: a scoping review. BMC Health Serv Res 21, 4 (2021). <https://doi.org/10.1186/s12913-020-05979-9>
8. Barrows, David , et al. Public-private partnerships in Canadian health care: A case study of the Brampton Civic Hospital, OECD Journal on Budgeting, 2012;12(1). http://dx.doi.org/10.1787/budget-12-5k9czxkbck9w
9. Forman R, Shah S, Jeurissen P, Jit M, Mossialos E. COVID-19 vaccine challenges: What have we learned so far and what remains to be done? Health Policy, 2021;125(5):553-567

   ,<https://doi.org/10.1016/j.healthpol.2021.03.013>.
10. Bollyky TJ. U.S. COVID-19 Vaccination Challenges Go Beyond Supply. Ann Intern Med. 2021;174(4):558-559. doi:10.7326/M20-8280
11. van Eeuwijk P, Angehrn Z. How to … Conduct a Focus Group Discussion (FGD). Methodological Manual. Available from: https://www.researchgate.net/publication/324075132_How_to_Conduct_a_Focus_Group_Discussion_FGD_Methodological_Manual [accessed Apr 04 2022].
12. The Cable. FULL LIST: Lagos opens 88 centres for COVID-19 vaccination. Latest Nigerian News. Available at: <http://www.latestnigeriannews.com/news/338062/> [cited 2021 Apr 7].
