# Supplementary material for: A qualitative analysis of the COVID-19 vaccination rollout in Lagos, Nigeria: Client and provider perspectives on the plan, the process and the progress
Source: PLOS Glob Public Health. 2022 Nov 8;2(11):e0000486. doi: 10.1371/journal.pgph.0000486 (PMC10021997; doi:10.1371/journal.pgph.0000486)
Supplement: Attachment — Submitted filename: Response to reviewers 2 -Aug 2022.doc [file pgph.0000486.s002.doc]

**Response to Reviewers**

**Title of manuscript: A qualitative analysis of the COVID-19 vaccination rollout in Lagos, Nigeria: Client and provider perspectives on the plan, the process and the progress**

| **Reviewer/**  **section** | **Original comments of the reviewer** | **Reply by the author(s)** | **Correction made** |
| --- | --- | --- | --- |
| **Reviewer 1** | Thank you for reviewing your paper and providing a useful table reflecting your responses. I think the paper is worthy of publication, I would simply make a few remaining recommendations to fully address the comments: | The reviewer’s comments are appreciated.  All corrections have been made. |  |
|  | The only significant comment is about the literature-- I think the authors still need to situate the study more firmly in the existing literature, they do so in the discussion but I think it would be even more convincing if this literature was introduced in the Introduction and then returned to in the Discussion. This may be better than simply referring to a "theoretical framework" that then is not summarised or explained. The authors could simply argue that they analysed their data based on their analysis of the literature (i.e. developed an analysis framework based on themes that emerge from the literature-- which you would have hinted at in the introduction) OR they could summarise in a bit more detail what the theoretical framework they used was. | This is noted | The framework is based on a literature review. ^6,7,10,16^ |
|  | Also, I emphasise again to be careful with the language-- you are not actually reporting evidence of misconceptions (i.e. nobody, at least from the data you report, actually told you that they believe the rumours but they think that misconceptions matter) but rather *perceptions* that misconceptions and vaccine hesitancy are significant. Just make sure that the language reflects this-- e.g. line 629 (but worth checking throughout) | This is noted |  |
|  | Smaller comments from my reading of the text: |  |  |
|  | Line 200: a justifiable, agreeable FGD guide—not sure what this means, would remove the adjectives | The adjectives have been removed. |  |
|  | Line 293—I wouldn’t have quotes where people are just confirming each other’s answer—just summarise here what they said. You want to bring in a quotation when it expresses well a point you are trying to make (this is done much better in the next paragraph for example) | This is noted. |  |
|  | Line 329: perceptions of benefits etc?; |  |  |
|  | 385 The health workers blamed misinformation for the very frequently seen vaccine hesitancy, -- I would still say “blamed misinformation for what they saw as vaccine hesitancy” (you are not actually reporting data on whether hesitancy has been high) | This is noted and has been corrected. | The health workers blamed misinformation for the very frequently seen behavoiur they perceived as vaccine hesitancy |
|  | Remove hesitations from the transcripts (can just put … instead of ehmmm) | This has been done. |  |
|  | 519: bottom-up approach for recommended for consideration in future vaccination planning – check grammar + who was it recommended by? |  | bottom-up approach was recommended for consideration in future vaccination planning  The administrators recommended that the grass-root citizens be carried along and their opinions included in the vaccination planning process describing a bottom-to-top approach. |
|  | 559- I wouldn’t list the challenges as if it waa a bulleted list—reads better if you frame it as a complete sentence “e.g. key informants reported what they termed psychological challenges…” (are these your terms?) |  | The challenges associsted with the COVID 19 vaccination were termed as social, psychological and economic |
|  | 629: the study does not in fact identify misconceptions but rather reports concerns about misconception |  | This study however reports concerns about identified misconceptions from misleading information about the side effects of the vaccine |
|  | 637—wonder whether you need to introduce some of the literature on vaccine deployment and confidence a bit earlier on in the intro to situate your study and then return to it in the discussion | This is noted |  |
|  | 665—what does grassroot citizens mean? Maybe just citizens | Grassroot’ has been removed |  |
|  | Overall:  - I would recommend a thorough proof read as there are some typos  - There are some inconsistencies in how you use transcription conventions—for anything that was not said verbatim but added as context, I would use square brackers  - This is up to the authors but I would remove hesitations from the transcripts (can just put … instead of ehmmm) | The paper has been proof-read  Changed to square brackets  This has been done |  |
|  |  |  |  |
| **Reviewer 2** | This is a revision of a paper focused on the COVID-19 vaccine rollout in Lagos, Nigeria. I think the authors should be commended for their work in improving the original manuscript. The topic remains interesting and important, and this version is presented much clearer. Well done! | The reviewer’s comment is noted with thanks. |  |
|  | I think there are still some small changes, mostly just clarifications, that would make the paper even stronger. Those suggestions are included below: | This is noted. |  |
|  | **Introduction** |  |  |
|  | L 90 – What is an “epidemiological end”? | The sentence has been rephrased for clarity. | Successful vaccine rollout would interrupt the natural spread of the virus and lead to an end to the COVID-19 pandemic |
|  | L 93-94 – Mention the location of the first mass vaccination program; Can you also give more specific information for “the number of doses of the vaccine available”? | This is noted and corrections have been made. | The first mass vaccination programme started in the UK in early December 2020  At the time of writing, the number of doses of the COVID-19 vaccines secured globally was given as 17.5 billion |
|  | L 101 – is “discredited” the right word to use? Perhaps something more about the vaccine being found safe instead? | This is noted and corrections have been made. | However, these reports about the formation of blood clots in recipients of the vaccine have since been proven to be false |
|  | L 104 – capitalize the W in World | This is noted and the correction has been made. |  |
|  | L 106 – Please put a citation after the 1% | This is noted and the correction has been made. |  |
|  | L 108 – What do you mean by “seem to be reducing”? Can you be more specific? Is Africa receiving fewer vaccines or are more vaccines being given to other regions? | The provision of vaccines for Africa were irregular and sometimes experienced delays and it appeared to have been reduced. |  |
|  | L 109-111 – What was exemplary about Botswana, Ethiopia, and Ghana’s rollouts? Was it the speed? The proportion of the population reached? | It was a mixture of all these. |  |
|  | L 148 – What was the amount of time between the vaccines arriving in country and making all adults over 18 eligible? | Adults over 18 became eligible to be vaccinated when all those who were given priority had been vaccinated and this was based on the situation in different locations. There is no specific date. |  |
|  | **Methods** |  |  |
|  | General: The authors provide a lot of information on their methods, which I really appreciate and find it makes it clear as to what they did as part of the study. I think, in general, the structuring of the methods section can be cleaned up. For example, the information in the sub-section “data collection tools and techniques” can be placed in each of the sections on the specific tools. That way all the information on the FGDs, KIIs, and IDIs is together and does not need to be repeated. This will make it easier to follow. | This is noted with thanks |  |
|  | L 158 – Please add the abbreviations in parenthesis, (LGA) and (LCDA) as they are abbreviated later on in the methods section | This is noted and the correction has been made. |  |
|  | L 164 – do you mean 88 primary health facilities? | A few General Hospitals were included. | However, the COVID-19 vaccination in Lagos State was conducted in 88 health facilities across the state, which included PHCs and General Hospitals. |
|  | L 167 – How many of the COVID-19 vaccination centres were involved in the study? Was it all 88 (mentioned above)? |  | L 180 - 7 LGAs from the urban LGAs and 1 LGA from the rural LGAs were selected by ballot, a total of 8 LGAs. |
|  | **Results** |  |  |
|  | L 260 – Perhaps Table 1 could also include the gender and age breakdown of participants? What is the median age of the different groups? | Age and gender were collected for only the FGD participants and not frontline workers or the administrators. Adding the age and gender details of the FGD participants to table 1 makes the table appear incomplete and it was advised by reviewers to leave those details in the text. |  |
|  | L 277-281 – I understand wanting to use quotes, but I think they are more powerful elsewhere. Here, this information could be condensed to something like “clients had differing beliefs on the age for eligibility, ranging from 15 years and above to twenty years and above.” In this way, the following quotes about clients’ worry on the sustainability of the vaccine can be better highlighted, as I find that a very interesting and important finding.” |  | for example in FGD 1 (Ikorodu), clients had differing beliefs on the age for eligibility, ranging from 15 years and above to twenty years and above. |
|  | L 349 – The follow up after the quote is not really a result – it’s more of a discussion… | “a genuine concern” has been deleted | However, IDI 1 expressed concerns saying, “I think the disadvantage {of being vaccinated} is that some people that are vaccinated believe they are fully covered 100%, they don’t want to use their face masks anymore, they don’t want to wash their hands regularly, they don’t want to use their hand sanitizer, they believe they can just go everywhere without face mask”, a genuine concern, given that hand hygiene and face coverings are required even after vaccination for the control of the pandemic. |
|  | L 369 – Was this scoring a formal part of the IDIs (e.g. a given scale that was asked of all participants), or was it asked as a general question to gauge participants’ view? If the former, it should be mentioned in the methods section. Otherwise, perhaps it could be written more general… | It was asked as a general question to gauge participants’ view. | The scoring of the vaccination programme, when the frontline workers were asked as a general question to gauge their view, was mostly above average. |
|  | L 399 – the key informants gave the strengths of the plan and those are reported – did they have anything they thought was perhaps a weakness of the plan or a gap in the plan? | Yes. The weaknesses were captured there by ID1 17 |  |
|  | L 411 – Did the respondents give any thoughts on the 4 stages of rollout more specifically? | No |  |
|  | L 440 – I like the inclusion of what people did when they experienced challenges in the process. Where there any other examples of challenges that people had to overcome? | All the challenges were reported here. |  |
|  | L 472 – Did key informants give specifics as to what they thought needed more funding? Or to what they thought did not have enough funding? How did they overcome challenges in funding? | The whole vaccination exercise was said to need more funding. They were not in the position to influence funding. |  |
|  | L 487 – Was this support given by the government/government employees or by the community members? | The support was given by the Local Government. |  |
|  | General - The barriers caused by internet access is mentioned several times in the paper and it’s mentioned that paper records were used instead. As this seems like a big issue, was there anything else mentioned by study participants about what they did to overcome the challenges of internet? How did it affect their work? How do they suggest this is altered for future vaccine efforts? | Paper records were the only measures taken to overcome the problem of poor internet supply. Their work had to continue using paper records.  Better internet provision was the obvious solution. |  |
|  | Table 2 – Perhaps more detail in last strategies cell for Provider Perspectives (“Better planning backed by correct data” – was there anything more specific?) | Better planning backed by correct and accurate data. |  |
|  | **Discussion** |  |  |
|  | General – I got a little lost sometimes in the Discussion regarding whether or not cited studies or examples were for COVID-19 or vaccines in general, or if they were taking place in the study sites, Nigeria, Africa, or elsewhere. Just make sure you’re more clear as to where/what this information references. | This is noted with thanks |  |
|  | L 586 – where did this study take place? | Nigeria |  |
|  | L 603-605 – Do you mean this about vaccines in general, or for COVID specifically? | vaccines in general |  |
|  | L 605 – What is “very impressive”? Can you provide more specifics? | It means that some immunization sites had quite remarkable vaccination turnout |  |
|  | L 608 – This is another area that would benefit from some more specific information. For example, what is poor turnout? Are these sites that were included in the study, or elsewhere? | Poor turnout refers to a small number of vaccine recipients.  This was referring generally to the vaccine sites in Lagos. |  |
|  | L 615 – Did recommendations like local language come up in the FGDs with the clients? If so, that should be made more clear in the results. | It did not come up |  |
|  | L 627 – “enablers of poor vaccine uptake” should be changed to “challenges (or barriers) to vaccine uptake” |  | Aside from health system-centered challenges, clients reported barriers to vaccine uptake, notably, AEFIs. |
|  | L 637-640 – Can you provide more information on what you mean by “bottom-to-top”? Not the concept, but how this relates to vaccine rollout strategies and how that would compare to the current strategy assessed in the study. |  | Participants recommended that the grass-root citizens be carried along and their opinions included in the vaccination planning process |
|  | L 643-649 – This seems like a strong recommendation. Are there any examples of other places that have done this, either for COVID or for other vaccines, that can be used as an example? | This example was referenced  ^50^ Marathe A, Lewis B, Barrett C, Chen J, Marathe M, Eubank S, et al. (2011) Comparing Effectiveness of Top-Down and Bottom-Up Strategies in Containing Influenza. PLoS ONE 6(9): e25149. <https://doi.org/10.1371/journal.pone.0025149> |  |
|  | General – I think it is in the flow of the Discussion, but for the sake of continuity, I think keeping the plan-process-progress component in the Discussion could be helpful. | This is noted. Corrected. |  |
|  | General – I also think there could be a bit more attention paid to where clients, frontline workers, and key informants agreed and disagreed. It sounds like they mostly agreed, so what may that mean for the recommendations that are made? | This has been corrected. |  |
|  | General – I think one thing that could be interesting to highlight more is how challenges were overcome by the frontline workers. Many of the challenges will be universal, so to see how they were dealt with could be helpful to other countries or institutions rolling out vaccine campaigns. | This is noted. All the actions taken to overcome the challenges that were reported have been included in the manuscript. |  |
|  | General - What were the results of the vaccine rollouts in the study sites? Were they in line with the rest of the state/country? If not, how may this have influenced the results? To keep anonymity, I don’t think the specific numbers need to be given, but it would be helpful to know how the study sites compared to other sites. | Records of the vaccine rollouts in other states were not available. |  |
